# Sphingosine Kinase 1 in Breast Cancer—A New Molecular Marker and a Therapy Target

**DOI:** 10.3389/fonc.2020.00289

**Published:** 2020-03-20

**Authors:** Heba Alshaker, Hannah Thrower, Dmitri Pchejetski

**Affiliations:** ^1^School of Medicine, University of East Anglia, Norwich, United Kingdom; ^2^Faculty of Medicine, Imperial College London, London, United Kingdom

**Keywords:** sphingolipids, sphingosine kinase 1, breast cancer, progression, chemoresistance, targeted therapy, molecular marker

## Abstract

It is now well-established that sphingosine kinase 1 (SK1) plays a significant role in breast cancer development, progression, and spread, whereas SK1 knockdown can reverse these processes. In breast cancer cells and tumors, SK1 was shown to interact with various pathways involved in cell survival and chemoresistance, such as nuclear factor-kappa B (NFκB), Notch, Ras/MAPK, PKC, and PI3K. SK1 is upregulated by estrogen signaling, which, in turn, confers cancer cells with resistance to tamoxifen. Sphingosine-1-phosphate (S1P) produced by SK1 has been linked to tumor invasion and metastasis. Both SK1 and S1P are closely linked to inflammation and adipokine signaling in breast cancer. In human tumors, high SK1 expression has been linked with poorer survival and prognosis. SK1 is upregulated in triple negative tumors and basal-like subtypes. It is often associated with high phosphorylation levels of ERK1/2, SFK, LYN, AKT, and NFκB. Higher tumor SK1 mRNA levels were correlated with poor response to chemotherapy. This review summarizes the up-to-date evidence and discusses the therapeutic potential for the SK1 inhibition in breast cancer, with emphasis on the mechanisms of chemoresistance and combination with other therapies such as gefitinib or docetaxel. We have outlined four key areas for future development, including tumor microenvironment, combination therapies, and nanomedicine. We conclude that SK1 may have a potential as a target for precision medicine, its high expression being a negative prognostic marker in ER-negative breast cancer, as well as a target for chemosensitization therapy.

## Breast Cancer-current Trends

Over the last 50 years, the breast cancer profile has changed enormously, with more women surviving the disease than ever before. Since the 1970s, the incidence of female breast cancer in Europe has increased by 72%, and it is now the most common cancer in women in the UK (1). Despite this, mortality has fallen dramatically over the last 40 years, with the 5-year survival for women diagnosed with breast cancer reaching 87% (1). However, it is still the second major cause of cancer death for women in the UK, second only to lung cancer (1). Therefore, it is imperative to investigate the mechanisms of breast cancer progression in order to identify new molecular targets for treatment and subsequently new therapies.

There are many risk factors associated with the development of breast cancer, including age, female sex, family history, increased body mass index (BMI) (2–4), and various reproductive factors related to lifetime levels of sex hormones, including: early menarche; late menopause; nulliparity; hormone replacement therapy, and lactation (which is protective against breast cancer) (5). In addition, there are genetic factors that can dramatically increase the risk of breast cancer, the commonest being mutations in the DNA maintenance and repair genes, BRCA 1/2, which confer a 60–80% risk (6).

In the UK, the NHS breast screening program offers mammography to all women aged 47–73 (7). If patients present with symptoms suggestive of malignancy, they are referred to a specialist breast unit within 2 weeks to undergo a triple assessment (clinical history and examination, imaging, and biopsy) (8). Similarly, if a lesion is seen on imaging, such as screening mammography, a core needle biopsy will be taken to provide a diagnosis (9), and if indicative of breast cancer, tissue is sent for further examination to determine hormone (estrogen and progesterone) receptor and human epidermal growth factor (HER2) status, as this will influence treatment decisions and provide information regarding prognosis (10).

Breast cancer subtypes are defined using different approaches. In the past, it was classified according to histological type and grade, with the later addition of hormone receptor and HER2 status (11). Histologically, breast cancer can be divided into *in situ* (ductal and lobular) and invasive cancer, of which there are over 20 different types (12). The most common is invasive ductal carcinoma, which makes up 75% of cases of breast cancer, followed by invasive lobular carcinoma, comprising 10% of the cases (13). Tumors are assigned one of three grades, with grade 1 being well-differentiated and grade 3 being poorly differentiated (14, 15). Tumors are staged using the TNM (tumor, node metastasis) system (12, 16).

As described above, after histological examination, tissue samples are analyzed to identify the presence, or absence, of hormone receptors (estrogen and progesterone) and HER2 status (17). Expression of these receptors influences treatment decisions as the presence of the estrogen receptor (ER), expressed in ~80% of breast tumors (18), determines a tumor's response to endocrine therapy while expression of HER2 (19) means that the cancer can be treated with monoclonal antibodies that specifically target this receptor, such as trastuzumab (herceptin) (20, 21). When all three markers are absent, the breast cancer is described as triple negative; this constitutes ~10–15% of breast tumors (11) and has the worst prognosis, with a more aggressive phenotype carrying an increased risk of recurrence (22, 23).

During the last 15 years, a classification system based on gene expression profiling has been developed, which offers more information about prognosis and can help to guide clinicians in decisions regarding therapy. It was first described in 2000 (24) and split breast cancer into four subtypes: luminal, HER2, basal-like, and normal-like. The former has since been divided into two (luminal A and B) (24, 25), and new categories are continually being added, such as the claudin-low and molecular apocrine subtypes (26–29). This mode of classification is increasingly being used in clinical practice, with several assays now available, the best known being Oncotype DX (30) and Mammaprint (31).

The two luminal subtypes are characterized by expression of the ER; luminal A tumors, comprising 50–60% of breast cancers, have low levels of expression of cell proliferation genes (24, 32), while luminal B tumors, which make up 10–20% of tumors, have high levels of these genes and confer a worse prognosis (33, 34). The two can be distinguished by levels of Ki67, a marker of cell proliferation (35). HER2 overexpressing tumors (15–25% of breast tumors) are characterized, evidently, by increased expression of HER2 and HER2-associated genes, as well as genes linked to cell proliferation (36), and carry a worse prognosis than the luminal subtypes; however, with the advent of targeted treatment, survival has improved dramatically (19, 20, 37, 38). Basal-like tumors are characterized by expression of genes usually present in myoepithelial cells and are often high grade and very aggressive, resulting in a poorer prognosis (39). Normal-like tumors make up 5–10% of breast cancers and are traditionally grouped together with other breast abnormalities, such as fibroadenomas and normal breast tissue samples (24); however, there is some debate over whether this class truly exists, as many believe that the samples that fall into this class simply contain high levels of normal breast tissue (40, 41).

The treatment of breast cancer requires a multidisciplinary approach; many therapeutic modalities are available, with the choice of treatment depending on the presence of certain markers and tumor staging (9). Generally speaking, patients with early-stage breast cancer will be offered breast conserving surgery with adjuvant radiotherapy, with mastectomy offered when breast conserving surgery is not suitable or when chosen by the patient (8), both of which have equivalent survival rates (42). Often, medical neo-adjuvant therapy is given to patients prior to surgery to reduce tumor size (8). Management of the axilla must also be considered; when a diagnosis of breast cancer is made, axillary staging is performed by ultrasound and cytology or core biopsy (8). Whereas in the past, radical axillary clearance was the norm, today, sentinel lymph node (SLN) biopsy is favored if the axilla is clinically negative (43). However, the best management for patients with a positive SLN biopsy is still unclear as approximately half of patients who have a positive biopsy do not have further lymph node involvement (44), and there is evidence to suggest that axillary radiotherapy as opposed to complete axillary clearance would be equally effective in eradicating disease in the axilla (45, 46).

Decisions regarding post-operative adjuvant therapy are dependent on many factors, including the tumor stage, the grade and histological type, the expression of hormone receptors, and the HER2 and molecular subtype (24). Patients with ER positive cancer will be offered endocrine therapy: tamoxifen if premenopausal and aromatase inhibitors if postmenopausal (8), while HER2 positive tumors will be treated by biologic therapies involving monoclonal antibodies (9). Adjuvant chemotherapy has been shown to reduce the relative risk of death (47), but it is still unclear which patients will benefit. Radiotherapy, either whole breast or partial irradiation, can be used in several circumstances: post-lumpectomy (48); in patients with large (>5 cm) tumors; in those with four or more positive lymph nodes; for tumors with close margins; and for inflammatory breast cancer (49).

For advanced breast cancer, medical therapy is the mainstay of treatment and aims to improve survival while maintaining a good quality of life. Choice of medical treatment depends on hormone receptor and HER2 expression, with ER positive tumors treated with endocrine therapy and HER2 positive tumors treated using monoclonal antibodies. Chemotherapy is given in a number of circumstances including breast cancer that is resistant to hormonal therapy and hormone-receptor negative, HER2 positive, or rapidly progressive breast cancer (8). In addition to the treatment of the primary tumor, patients will also require therapy to control metastases, such as bisphosphonates for bone metastases (50) or radiotherapy for brain metastases (8).

Several signaling pathways have been implicated in the development of breast cancer; one well-known example is the HER2 pathway, alterations in which can result in sustained proliferation signaling and cell survival (36). The HER2 receptor is a tyrosine kinase receptor, which, when activated, forms dimers within the plasma membrane to activate three major signaling pathways: Ras/Raf/MAPK, JAK/signal transducer and activator of transcription (STAT), and PI3K/AKT/mTOR (51, 52), which control various aspects of cellular biology, including cell growth, proliferation, division, metabolism, migration, survival, and apoptosis.

Another pathway associated with breast cancer is the insulin-like growth factor 1 receptor (IGFR1)/PI3K/AKT/mTOR pathway (51, 53). There is evidence to suggest that overexpression of IGFR1 can lead to the development of tumors and promote formation of metastases (54). Mutations can occur at several points along this pathway, enhancing tumor development, and cancer cell survival; for example, PI3K mutations have been found in up to 25% of breast cancers and up to 35% of ER-positive cancers (55). Such mutations are thought to play a role in resistance to treatment (52), and mutations in inhibitors of this pathway, such as PTEN, have also been implicated in breast cancer (56). It has been proposed that targeting the adenosine monophosphate kinase pathway, which opposes the IGFR1 pathway (57, 58), may prove to be effective in the treatment of breast cancer.

The pathways that regulate angiogenesis are also important targets in the search for breast cancer treatments. One in particular is the vascular endothelial growth factor (VEGF) pathway, which has been the focus of much research in recent years, as it appears to be the most important pathway controlling angiogenesis in the first stages of cancer development. Moreover, it has been shown that the addition of the monoclonal antibody bevacizumab, a VEGF inhibitor, to chemotherapy regimens in HER2 negative breast cancer and in triple negative cancer significantly increases progression-free survival, as well as increases overall survival in the triple negative group (59).

## Sphingolipid Signaling in Cancer, a Brief Summary

Sphingolipids are a class of lipid molecules involved in the structure of the eukaryotic plasma membrane (60). In recent years, they have increasingly been the focus of attention, having emerged as cell signaling molecules and involved in normal physiology as well as cancer cell pathophysiology (61). Sphingolipid metabolism is complex and generates an array of molecules; three of these molecules, namely, ceramide, sphingosine, and sphingosine-1-phosphate (S1P), act as signaling molecules and are involved in many biological processes within the cell, controlling survival, proliferation, differentiation, and apoptosis (62). Ceramide sits at the center of sphingolipid metabolism and can be converted to the pro-apoptotic sphingosine by ceramidase. Sphingosine can further be metabolized to form the anti-apoptotic S1P by the action of sphingosine kinases (SKs), of which there are two human isoforms, SK1 and SK2. The balance between levels of ceramide and S1P is thought to be central to determining whether a cell survives or undergoes apoptosis (63).

The production of S1P through the action of SK1 activates several pathways within the cell by the binding of S1P to one of five G-protein-coupled receptors on the plasma membrane (64). These receptors are expressed in varying levels in different tissues, and upon the binding of S1P to its receptor, a variety of downstream signaling cascades can be activated (65), promoting actions including cell proliferation and migration, activation of the inflammatory response, fibrosis, angiogenesis, nociception, and inhibition of apoptosis. S1P may also be able to regulate the same intracellular processes independently of a receptor (66); several mechanisms have been proposed, including the binding of S1P to histone deacetylases 1/2, resulting in epigenetic gene expression (67).

In this way, SK1 also has a role in cancer progression, facilitating many properties of cancer cells, including oncogenic transformation (68), tumor growth (69), impairment of apoptosis (70), tumor vascularization (71), and metastatic spread (72). Furthermore, high SK1 levels correlate with poor prognosis and reduced survival time in many cancers (62, 73, 74). SK1 also plays a crucial role in resistance to cancer therapy, and targeting the SK1/S1P pathway has been proven to be effective in the treatment of various cancers (62, 75).

In a recent meta-analysis (74), SK1 was shown to be significantly associated with several types of cancer, including breast, lung, ovarian, gastric, and kidney. Significant differences in SK1 expression were found between cancer tissues, adjacent non-cancer tissues, and benign tissues; these results are suggestive of a gradual increase in SK1 levels from benign to cancerous cells. This study examined the expression of SK1 mRNA and protein in cancer cells, which demonstrated increased levels when compared with normal cells. Finally, in terms of survival, higher rates of SK1 expression correlated with reduced 5-year and overall survival (74).

Several studies involving knockout mice have contributed to current thinking that SK1 can be considered as a proto-oncogene; for example, the size of multiple intestinal adenomas was reduced in response to SK1 knockout (76). In addition, other studies have shown that SK1 knockout is protective against the development of colon cancer (77, 78), and similar results have been produced with other cancers, such as head and neck squamous cell carcinoma (79), lymphoma, and osteosarcomas (80).

Expression of SK1 can be upregulated through the action of several first and second messengers, including growth factors, cytokines, receptor tyrosine kinases, and toll-like receptors; this process of upregulation of SK1 expression varies depending on the type of cancer (81, 82). Release of such cell signaling mediators stimulates the phosphorylation of SK1 by extracellular-regulated kinase (ERK1/2) (83) and protein kinase C (PKC) (84), initiating various signaling cascades within the cell, resulting in cell survival, and proliferation. Additionally, SK1 expression can also be influenced by hormones (75), an interaction shown to be true in several types of cancer, including breast (85, 86), prostate (87), and neuroblastoma (88).

As well as production of S1P, SK1 may have the ability to regulate cellular processes through its interaction with other signaling proteins (62). Some interactions, such as those described above, increase the activity of SK1, while others result in the upregulation of other proteins to further enhance cell survival. Furthermore, it has been suggested that these interactions may have an effect on the clinical outcome (89). Additionally, increased activity of SK1 leads to decreased levels of the pro-apoptotic molecule, sphingosine, preventing it from switching off anti-apoptotic signaling, thus promoting cell survival (90).

SK1 has also been touted as a potential prognostic marker (73, 75, 91), as, in several cancers, higher SK1 levels correlate with higher-grade tumors, reduced survival times, and faster recurrence times. However, other reports found no correlation between SK1 expression alone and disease outcome (92), suggesting that either patient stratification or the mode of SK1 assessment (RNA vs. protein) may be critical for establishing meaningful clinical correlation.

## Sphingosine Kinase 1 and Breast Cancer

### Role of SK1 in Breast Cancer Signaling—Rationale for Targeting SK1

**Figure 1:**
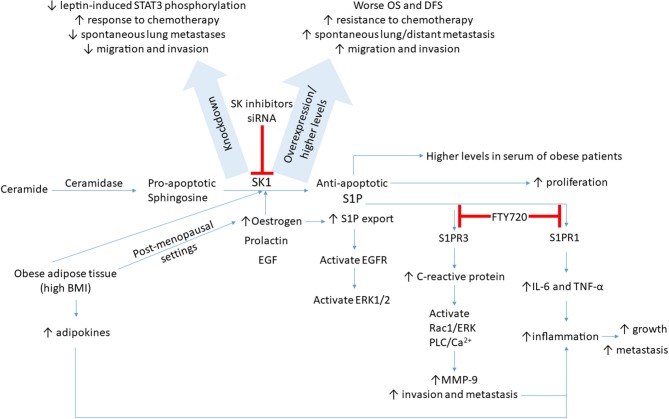
A schematic presentation of possible signaling pathways through which SK1/S1P axis is regulated in breast cancer. SK1/S1P axis impacts breast tumor growth, drug resistance, and metastasis (please see text for details). BMI, body mass index; DFS, disease-free survival; EGF, epidermal growth factor; EGFR, EGF receptor; ERK, extracellular-regulated kinase; IL-6, interleukin-6; MMP-9, matrix metalloproteinase 9; OS, overall survival; S1P, sphingosine-1-phosphate; STAT3, signal transducer and activator of transcription 3; SK, sphingosine kinase; TNF-α, tumor necrosis factor alpha.

Sphingolipid metabolism is deregulated in cancer cells in which SK1 and its product S1P have a critical involvement in a variety of biological responses (93) (summarized in Figure 1). In a comparison of five breast cancer cell lines and normal breast epithelial cells, it was found that the triple negative breast cancer cell line MDA-MB-231 had the highest levels of SK1 mRNA expression, protein expression, and enzyme activity. SK1 inhibition in these cells resulted in a decrease in cell proliferation and an increase in apoptosis, an effect not seen in non-cancerous breast epithelial cells and seen to a lesser extent in ER-positive breast cancer cell lines (94, 95).

Triple negative breast cancer is regarded as the most aggressive form of the disease. A recent analysis revealed that SK1 mRNA and protein expression is higher in triple negative breast cancer cell lines compared to non-triple negative breast cancer cell lines (96). SK1 appears to be a critical regulator of triple negative breast cancer metastasis. Overexpression of SK1 in MDA-MB-231 cells *in vitro* increased their migration and invasion without significant changes in proliferation (96). Orthotopic injection of MDA-MB-231 cells overexpressing SK1 into mammary fat pads of nude mice enhanced spontaneous lung metastasis (96). SK1 knockdown in another triple negative breast cancer cell line (MDA-MB-435) decreased their migration and invasion *in vitro*. Orthotopic injection of MDA-MB-435 with SK1 knockdown into nude mice decreased the number of spontaneous lung metastases (96).

Ingenuity pathway analysis identified the metastasis-promoting gene *FSCN1* as a top SK1-regulated gene. SK1 upregulates the transcriptional expression of FSCN1 through a nuclear factor-kappa B (NFκB)-mediated mechanism (96). FSCN1 exhibited an expression pattern similar to SK1; both were upregulated in basal subtype breast cancer compared with other subtypes, and correlated with a poor survival rate and increased distant metastasis in triple negative breast cancer patients (96).

In addition to metastasis and invasion, SK1 expression plays a role in breast cancer cells' survival. Cytotoxic effects of doxorubicin and fluorouracil in triple negative breast cancer cells (MDA-MB-231 and its variant LM2-4) were enhanced by silencing of SK1 (97). Functionally, SK1 was shown to regulate the levels of Notch signaling target gene Hes1 via S1P receptor (S1PR) 3-mediated upregulation of Notch intracellular domain (97). Collectively, pharmacological inhibition of SK1 appears to be an effective therapeutic strategy for the treatment of triple negative breast cancer (please see more details in the section *SK1 Inhibition as a Therapeutic Tool—Preclinical Evidence*).

S1P induces the proliferation of human ER-positive and ER-negative breast cancer cells (98) and enhances the survival and anchorage-independent growth of ER-positive MCF-7 cells (99). SK1 overexpression in MCF-7 cells led to more aggressive and larger tumors in nude mice (99) and promoted resistance to tamoxifen, which could be restored by silencing of SK1 (100). Indeed, in ER-positive human patients, high cytoplasmic expression of SK1 in breast tumors is associated with increased resistance to tamoxifen, and reduced patient survival and recurrence time (101, 102), which suggests that SK1 plays a role in hormone resistance.

Estrogen induces SK1 expression in MCF-7 breast cancer cells in a dose-dependent manner and produces a biphasic pattern (85, 103, 104). The first peak is thought to be mediated by non-genomic estrogen signaling, while the second is likely to be due to genomic effects, secondary to the binding of estrogen to its nuclear receptor, as evidenced by a reduction of only this peak in response to the addition of a transcriptional inhibitor (103). Similarly, estrogen produced a biphasic upregulation of SK1 activity and downregulated microRNA (miR)-515-5p in MCF-7 cells; miR-515-5p subsequently directly interacted with SK1 3′-UTR and regulated its expression (104). A similar biphasic pattern is seen when MCF-7 cells are treated with prolactin (85). Epidermal growth factor (EGF) also has the ability to stimulate SK1 activity within MCF-7 cells (105, 106). Again, a biphasic response is seen (105), with the second peak dependent on *de novo* protein synthesis. Treatment of cells with EGF induces migration of SK1 to the plasma membrane, particularly toward lamellipodia, within the first minute (106), which enhances cell motility, growth, and apoptosis stimulated by EGF. Various signaling pathways are involved in this process, namely Ras/MAPK, PKC, and PI3K (105). Although several ligands are able to upregulate the SK1 activity, it appears that only estrogen has the ability to induce S1P export from MCF-7 breast cancer cells (107).

There have been many studies that evaluate the link between estrogen, SK1, S1P, and EGF receptor (EGFR). Both estrogen and S1P have the capacity to activate EGFR (108). In MCF-7 cells, localized measurement of EGFR levels in response to EGF showed an EGFR decrease at the plasma membrane with a concurrent transient increase in endosomal levels. When stimulated by estrogen or S1P, EGFR levels at the plasma membrane also fell but at a slower rate than when activated by EGF, and endosomal levels increased over the time course of the experiment (109). Downstream of the receptors, the authors found that ERK1/2 was activated in response to all three ligands, but with estrogen and S1P, this activation was longer and more sustained. Similarly, activation of Cdc42 (one of the Rho GTPase family) was also longer, meaning that the internalization and degradation of EGFR are inhibited for longer in cells treated with estrogen or S1P.

S1P has been closely linked to inflammation in breast cancer. It has been shown to upregulate the expression of matrix metalloproteinase 9 (MMP-9) (110), which has been linked to tumor invasion and metastasis (111). Indeed, siRNA knockdown of MMP-9 significantly reduced the invasive and migratory phenotype of MCF-10A breast epithelial cells treated with S1P. Additionally, Kim et al. (112) have demonstrated that binding of S1P to S1PR3 was able to upregulate C-reactive protein expression in MCF-10A cells in a dose-dependent fashion. This, in turn, resulted in the activation of Rac1/ERK and PLC/Ca^2+^ signaling pathways, increasing MMP-9 expression, which, in turn, stimulated breast cancer invasion and contributed to the inflammatory environment (112).

Obesity plays a significant role in breast cancer pathogenesis. Estrogen production by adipose tissue (both local and systemic) is a well-established mechanism that contributes to breast cancer incidence and prognosis, especially in postmenopausal women (113, 114). In addition, adipose tissue is known to produce a wide variety of factors collectively termed adipokines, some of which have been shown to propagate breast cancer growth (115). Furthermore, obesity is known to induce a state of chronic inflammation in mammary tissue, which leads to breast cancer progression (116, 117). Sphingolipid signaling has previously been linked with the inflammatory response (118), and S1P serum levels positively correlate with BMI (119). In treatment-naive breast cancer patients, S1P levels were significantly higher in the serum of obese patients than in that of non-obese patients (120). Breast cancer patients with higher BMI also had higher SK1 mRNA levels in their tumors (86) and trended toward worse overall survival and disease-free survival (119). Mice fed with a high-fat diet had higher levels of S1P in the primary tumor itself, in tumor interstitial fluid (representative of the tumor microenvironment), in the systemic circulation, and in the lungs (representative of distant sites) (120). Moreover, the expression of SK1 and S1PR1 is higher in metastatic lesions, along with increased levels of pro-inflammatory cytokines, such as interleukin-6 (IL-6) and tumor necrosis factor alpha (120). As such, the SK1/S1P/S1PR1 axis is potentially implicated in obesity-related inflammation. FTY720 (fingolimod), a functional antagonist of S1PRs, successfully interfered with this feed-forward amplification loop. It was shown to reduce S1P levels and SK1 and S1PR1 expression in the breast tumors, as well as reduce key proinflammatory cytokines, macrophage infiltration, and tumor progression induced by obesity (120).

Leptin is a hormone involved in appetite regulation. Interestingly, its intratumoral levels were positively correlated with the worse outcome in breast cancer patients. A strong correlation between SK1 and functional leptin receptor expression was reported in human primary breast tumors and their associated lymph node metastases (86). The expression of SK1 and functional leptin receptor was elevated in metastases of ER-negative patients and showed a significant increase in the tumors of patients with higher BMI (86). In ER-negative breast cancer cells, SK1 knockdown significantly reduced leptin-induced STAT3 phosphorylation. Knockdown of another known activator of STAT3 signaling, glycoprotein (gp)130, also resulted in a significant decrease in leptin-induced STAT3 phosphorylation. Leptin-induced STAT3 is partially cross-activated through SK1-mediated IL-6 secretion and gp130 activation (121).

Drug resistance is an important factor implicated in the failure of breast cancer treatment. Analysis of gene expression in doxorubicin-treated patients showed higher expression of SK1, S1PR1, and other genes with a known role in the inflammatory process (such as STAT3, IL-6, and NFκB) following treatment (119). S1P functional antagonist and SK1 inhibitor FTY720 in combination with doxorubicin is capable of suppressing inflammation induced by doxorubicin. This combination inhibited growth of E0771 cells (a mouse mammary adenocarcinoma cell line expressing the ER) *in vitro* and suppressed the expression of S1P signaling-related genes, STAT3 and IL-6, as well as reducing tumor burden *in vivo* (119), suggesting that the SK1/S1P/S1PR1 axis plays a role in doxorubicin resistance. It has been shown that in triple negative breast cancer cell lines, MDA-MB-231 and BT-549, mTOR inhibitor RAD001 (everolimus) reduced SK1 expression and sensitized these cells to low-dose (5 nM) docetaxel (122). Furthermore, in an ER-positive patient cohort, higher BMI was positively correlated with the S1P signaling pathway but negatively correlated with the doxorubicin-resistant gene set, suggesting that the FTY720/doxorubicin combination may be particularly useful for ER-positive tumors in obese patients (119). Likewise, in triple negative breast cancer cells, FTY720 provided chemosensitization to docetaxel following encapsulation in nanoparticles allowing a 4-fold reduction in the effective dose and reduced chemotherapy-induced side effects (123).

### Patterns and Significance of SK1 Expression in Breast Tumors

It has been shown that in patients with breast, colon, lung, ovarian, gastric, uterine, kidney, and rectal tumors, there is at least a 2-fold increase in SK1 expression in cancer cells compared with normal tissues from the same patients (124). Table 1 provides an overview of SK1 expression patterns in human breast tumors and its clinical significance. In breast tumors, SK1 mRNA expression was shown to increase through the four stages of breast cancer and was associated with disease progression (93). High levels of SK1 correlated with poorer survival and prognosis in breast cancer patients (97). In patients with invasive ductal carcinoma, high SK1 expression was an independent factor for predicting shorter recurrence-free survival and was significantly associated with more aggressive oncogenic behavior, including higher histological grade, development of distant metastasis, negativity for estrogen, progesterone and HER2 receptors, and triple negativity (91). Triple negative breast cancer cells (97) and basal-like subtypes (which often lack the ER) exhibited the highest SK1 gene expression among the various molecular subtypes (95–97). In a study of 65 ER-negative tumors from patients with locally advanced or metastatic breast cancer who were receiving doxorubicin or docetaxel-based chemotherapy, it was shown that the tumors that failed to respond to chemotherapy exhibited significantly higher levels of SK mRNA compared to tumors that partially or completely responded to treatment (95).

**Table 1:** SK1 expression patterns and clinical significance in human breast tumors.

**Findings**	**mRNA/Protein (method, sample)**	**Reference**
Mixed cohort (*n* = 171 tissue samples, *n* = 1,098 microarray data): – Higher SK1 expression in ER-negative tumors – High SK1 expression in ER-positive patients insignificantly correlated with worse prognosis	mRNA (microarray, breast tumor tissue)	(125)
ER-positive patients treated with tamoxifen (*n* = 304): – High cytoplasmic SK1 expression is associated with a shorter mean time to recurrence on tamoxifen and a reduced mean disease-specific survival time – In ER-positive and HER1-3 positive tumors, high cytoplasmic SK1 expression is associated with an increase in the mean disease-specific patient survival time	Protein (IHC, FFPE tissue)	(101)
ER-positive patients treated with tamoxifen (*n* = 304): – Nuclear SK1 expression is associated with shorter time to recurrence on tamoxifen and shorter disease-specific survival – High levels of cytoplasmic SK1 and cytoplasmic ERK1/2 are associated with shorter time to recurrence on tamoxifen – High membrane S1PR1 expression is associated with shorter time to recurrence – High cytoplasmic S1PR3 expression is associated with shorter disease-specific survival – Membrane and cytoplasmic S1PR3 expression correlated with PR status and nuclear S1PR3 correlated with tumor size	Protein (IHC, FFPE tissue)	(102)
ER-negative (*n* = 140): – High SK1 expression is associated with shorter disease-specific survival in HER2-positive tumors – High cytoplasmic tumor S1PR4 is associated with shorter disease-free and disease-specific survival – High SK1 expression in tumors with low level of S1PR4 is associated with shorter disease-free and disease-specific survival	Protein (IHC, tissue microarray)	(126)
ER-positive patients treated with tamoxifen (*n* = 304): – High co-expression of nuclear SK1 and plasma membrane S1PR1 is associated with shorter disease-specific survival – High levels of both nuclear SK1 and cytoplasmic S1PR3 are associated with decreased mean disease-specific survival – High levels of either cytoplasmic or nuclear phosphorylated NFκB (p65) and nuclear SK1 correlate to shorter disease-specific survival and recurrence times – High expression of cytoplasmic phosphorylated c-Raf-1 or SFK or LYN or ERK1/2 or AKT and nuclear SK1 is associated with shorter disease-specific survival and recurrence time	Protein (IHC, FFPE tissue)	(89)
Mixed cohort (*n* = 112): – Higher SK1 expression in ER-negative tumors – Higher pathological complete response rate for tumors with high SK1 expression within the ER-positive luminal subtype of tumors – No correlation between HER2 status and expression of SK1 – No significant prognostic differences between tumors with high or low SK1 expression	Protein (IHC, tissue microarray)	(127)
Mixed cohort (*n* = 32 tissue samples, *n* = 3,992 microarray data): – Basal-like subtype had the highest SK1 gene expression among the various molecular subtypes – SK1 expression level is inversely correlated with overall and progression-free survival – Higher SK1 mRNA levels associated with no response to doxorubicin and docetaxel	mRNA (microarray, tumor tissue)	(95)
Invasive ductal carcinoma (*n* = 224) and ductal carcinoma *in situ* (*n* = 35): – High SK1 expression is correlated with higher histological grade, development of distant metastasis, HER2-, estrogen-, and progesterone-negativity, and triple negativity – Higher pathological T stage, higher pathological N stage, PR negativity, and high SK1 expression closely associate with distant metastasis in patients with invasive ductal carcinoma – Higher pathological T stage, lymph node and distant metastasis, advanced stage, lymphovascular invasion, progesterone-negativity, triple negativity, and high SK1 expression predicted poor overall survival in patients with invasive ductal carcinoma of the breast	Protein (IHC, FFPE tissue)	(91)
Mixed cohort (*n* = 236): – No significant relationship between SK1 expression alone and overall survival – No significance was observed for high vs. low SK1 protein expression alone following stratification for HER2 or PR – High SK1 inversely associated with both ER- and PR-positivity	Protein (IHC, FFPE tissue)	(92)
Mixed cohort (*n* = 65): – SK1 mRNA level is higher in breast cancer tissue compared to adjacent normal breast tissue – Basal-like subtype displays the highest SK1 gene expression – SK1 expression is higher in triple negative breast cancer patients – High expression of SK1 is correlated with poorer survival and prognosis – HER2-, estrogen-, and progesterone-negative tumors expressed higher SK1 mRNA	mRNA (RT-PCR, tumor tissue)	(97)
Triple negative breast cancers (*n* = 117): High expression of SK1 and FCSN1 correlated with increased distant metastasis and poor survival	Protein (IHC, FFPE tissue)	(96)

*ER, estrogen receptor; ERK, extracellular-regulated kinase; FFPE, formalin-fixed paraffin embedded; HER, human epidermal growth factor; IHC, immunohistochemistry; NFκB, nuclear factor-kappa B; PR, progesterone receptor; RT-PCR, real-time polymerase chain reaction; SK, sphingosine kinase; S1P, sphingosine-1-phosphate; S1PR, S1P receptor*.

SK1 is a constitutively active enzyme, which can also be phosphorylated, increasing its activity (83). Phosphorylation of SK1 was significantly associated with higher S1P levels in breast cancer tissue (97, 128), which correlated with lymph node metastasis (128). High SK1 expression was associated with a greater relative risk of development of distant metastasis compared to the risk of pathological T and N stages (91). These results potentially implicate SK1 as an important contributory factor in breast cancer spread. SK1 expression is a robust prognostic and predictive biomarker for the identification of patients at high risk of developing distant metastasis and shorter recurrence-free survival time (91).

SK1 expression has been demonstrated to be significantly higher in ER-negative tumors and is associated with poorer prognosis when compared to ER-positive tumors (95, 125). In another study, higher SK1 expression in ER-negative tumors was also observed; however, SK1 expression did not achieve a prognostic value for pathological complete response, which could be due to differences in the method of analysis (IHC compared to microarray gene expression profiling), smaller sample size of the latter study (968 samples compared to only 112 samples), or the fact that all patients in this study received tamoxifen (127). In ER-negative, HER2-positive breast tumors, high SK1 expression was significantly associated with reduced disease-free and disease-specific survival (126).

In ER-positive tumors, high cytoplasmic expression of SK1 is associated with increased resistance to tamoxifen and reduced patient survival and recurrence time (101, 102). Ohotski et al. (89) have shown that localization of SK1 in the nucleus of ER-positive tumors combined with either ERK1/2 or SFK or LYN or AKT or NFκB profoundly reduced disease-specific survival and recurrence times. Interestingly, not all S1PRs seem to have similar functions in breast tumor oncogenic signaling. High expression of S1PR1 and phosphorylated AKT or ERK1/2, as well as high expression of cytoplasmic S1PR3 and LYN, or nuclear S1PR3 and phosphorylated Raf1, were associated with shorter disease-specific survival time (89). By contrast, nuclear S1PR2 and c-Src were correlated with longer disease-specific survival time and reduced nuclear localization of SK1 (89), suggesting that S1PR2 counteracts the oncogenic action of SK1 and contributes to its translocation (73). Patients with triple negative breast cancer have high cytoplasmic SK1 and S1PR4 levels, which was shown to be associated with shortened disease-specific survival and recurrence times, as well as more advanced lymph node status, suggesting a role for both SK1 and S1PR4 in metastasis and as important prognostic markers in triple negative breast cancer (73). High cytoplasmic S1PR4 levels alone confer worse disease-free and disease-specific survival compared to tumors containing low levels of S1PR4 (126). It was also found that patients whose tumors contained high levels of SK1 and low levels of S1PR4 had shorter survival times compared to those with low levels of SK1, suggesting a functional link between the two.

Overexpression of HER2 increases SK1 expression and activity in MCF-7 breast cancer cells. In turn, this increase in SK1 expression reduces HER2 expression in a negative feedback manner, which limits the migration of these cells in response to S1P (101). Stratification of ER-positive patients according to the HER2 status showed that high cytosolic SK1 expression was associated with increased patient survival time and reduced recurrence rates in HER2 positive tumors (101). It was therefore suggested that for ER/HER2-positive breast cancer patients, the use of SK1 inhibitors might be detrimental (129). However, another study has shown that ER-positive patients with high SK1 and ERK1/2 expression had a shorter mean time to recurrence of 11 years (3 vs. 14) than patients with low SK1 and ERK1/2 expression, independent of progesterone receptor (PR), and HER2 status (102). Interestingly, in a recent study in ER-positive breast cancer, SK1 protein expression on its own had no correlation to overall survival or HER2/PR expression (92), suggesting that stratification of patients and the mode of SK1 assessment (RNA vs. protein) may be key for meaningful clinical correlation.

### SK1 Inhibition as a Therapeutic Tool—Preclinical Evidence

The evidence suggests that targeting SK has considerable therapeutic potential. Several inhibitors have been developed and tested in various cancer models, including the breast (Table 2). SK inhibitors can be broadly categorized into (a) pan-SK inhibitors (targeting both SK1 and SK2) and (b) inhibitors with more specificity toward a particular isozyme (we focused on SK1 in this review). Historically, pan-SK inhibitors were developed first (based on the sphingosine structure) followed by more isozyme-specific inhibitors, especially after the discovery of the SK1 structure in 2013 (135).

**Table 2:** Effects of SK inhibitors in breast cancer models.

**Inhibitor**	**Cell line/*in vivo* model**	**Observed effect**	**Reference**
**DUAL SK1/SK2 INHIBITORS (pan-SK INHIBITORS)**			
SKI-II	MCF-7	Blocked breast cancer viability, clonogenic survival, and proliferation and decreased estrogen signaling *in vitro*	(94)
SKI-II	MDA-MB-468, MDA-MB-231, MDA-MB-436/ MDA-MB-468 xenograft in mice	Inhibited triple-negative breast cancer cell growth *in vitro* and sensitized *in vivo* breast cancer xenografts to the EGFR inhibitor gefitinib	(130)
SKI-II	MDA-MB-231	Increased intracellular sphingosine, decreased PKC activity and cell proliferation, increased apoptosis	(131)
SKI-II	MDA-MB-453	Reduced basal and S1P/S1PR4-induced activation of ERK1/2 and modified HER2 trafficking	(126)
SKI-I	JC cell line (transformed murine mammary adenocarcinoma) allograft in BALB/c mice	Strong inhibition of tumor growth without overt toxicity	(132)
**SK1-SELECTIVE INHIBITORS**			
SK1-I	4T1-luc2 cell line (mouse mammary adenocarcinoma that expresses luciferase) allograft in BALB/c mice	Reduced the size and mitotic activity of the primary tumor, lymph node, and lung metastasis, and greatly decreased hem- and lymph-angiogenesis Reduced S1P levels in the tumor and in circulation	(133)
PF-543	MDA-MB-231	Impaired migration and invasion capability	(97)
SK1-5C	MDA-MB-231, MCF-7/ MDA-MB-231 xenograft in mice	Dose-dependent induction of growth arrest, increase in apoptosis, and inhibition of cell proliferation Decrease in serum-secreted S1P and serum-induced phosphorylation of both ERK1/2 and AKT in MDA-MB-231 Attenuated tumor growth in a mouse MDA-MB-231 xenograft model	(95)
SK-F	MDA-MB-231/ 4T1 allograft in BALB/c mice	Reduced cell proliferation Sensitized mouse breast tumors to docetaxel	(134)
FTY720	4T1 allograft in BALB/c mice	Chemosensitization to docetaxel, allowing a 4-fold reduction in the effective dose	(123)

*EGFR, epidermal growth factor receptor; ERK, extracellular-regulated kinase; PKC, protein kinase C; HER, human epidermal growth factor; S1P, sphingosine-1-phosphate; S1PR, S1P receptor*.

In JC transformed murine mammary adenocarcinoma allografts, pan-SK inhibitors (SKI-I and SKI-II) inhibited tumor growth without overt toxicity (132). The SK inhibitor, SKI-II (also known as SKi), has been shown to be effective in decreasing cell growth and survival of ER-positive MCF-7 cells (94), as well as decreasing ER-negative breast cancer cell growth *in vitro* (130). In addition, this inhibitor also decreased ERK phosphorylation, a known downstream effect of S1P signaling, and decreased transcriptional activity of the ER. A similar reduction in ERK1/2 activation following treatment with SKI-II was observed in ER-negative MDA-MB-453 breast cancer cells (126). Moreover, SKI-II has the ability to inhibit the actions of a tamoxifen-resistant ER, which highlights its potential for the treatment of endocrine-resistant breast cancer (94). In MDA-MB-468 xenograft tumors, SKI-II also demonstrated a chemosensitization effect when combined with gefitinib (EGFR inhibitor) (130). Similarly, combining SKI-II with paclitaxel resulted in an additive cytotoxic effect in MDA-MB-231 cells (131). Notably, pharmacologic inhibition of SK1 by SKI-II in MDA-MB-231 cells increased intracellular sphingosine (an endogenous inhibitor of PKC) levels, decreased PKC activity and cell proliferation, and caused accumulation of cells in S phase and SubG1 peak, indicating increased apoptosis (131).

In contrast to pan-SK inhibitors, the selective SK1 inhibitor SK1-I does not inhibit SK2 and several other protein kinases (136). SK1-I reduced tumor burden and metastatic growth of 4T1-luc2 tumors in mouse mammary fat pads by inducing tumor apoptosis, reducing SK1-produced tumor S1P levels, and reducing both tumor-induced hemangiogenesis and lymphangiogenesis (133). SK1 expression has been positively correlated with metastatic ability (96). Inhibition of SK1 using the selective SK1 inhibitor PF-543 (5 and 10 μM) impaired the migration and invasion capability of MDA-MB-231 cells *in vitro* and reduced the metastatic ability of MDA-MB-231 tumors in NOD/SCID mice (97). Interestingly, in head and neck carcinoma, this inhibitor lacked the potency to induce cancer cell apoptosis, despite a dramatic change in the cellular S1P/sphingosine ratio (137). The authors noted that this inhibitor did not seem to modulate cellular ceramide levels, which might explain why it failed in inducing cell death (137). Combining the SK1 inhibitor SK1-5C with doxorubicin and docetaxel significantly increased the cell death of MDA-MB-231 breast cancer cells (95). Clinically, tumors from patients with locally advanced or metastatic ER-negative breast cancer who failed to respond to doxorubicin or docetaxel-based chemotherapy had significantly higher levels of SK1 mRNA compared to tumors from partial or complete responders (95). Therefore, SK1 may have a potential as a prognostic marker in ER-negative breast cancer, as well as a target for chemosensitizing therapy (95).

A newer generation of inhibitors have been developed following the discovery of the SK1 crystal structure, which identified substrate binding pockets and protein binding domains (135). Co-crystallization of SK1 with PF-543 provided insight into improving SK1 selectivity (138). Compound 82 (139) [referred to as compound A (140)] was developed based on the crystal structure of sphingosine bound to human SK1. This compound was found to inhibit intracellular S1P production, both human SK1 and 2 isoforms, and mouse SK1, but not mouse SK2 (139). In contrast to docetaxel, this compound as a single agent failed to reduce the growth of MDA-MB-231 xenograft tumors (140), and no chemosensitization was attempted. Similarly, we found that our selective SK1 inhibitor compound SK-F (developed using field-template modeling) alone did not alter the *in vivo* growth of 4T1 (mouse triple-negative breast cancer cell line) cells. However, compound SK-F sensitized mouse breast tumors to subtherapeutic doses of docetaxel (134). Contrary to docetaxel, SK-F did not induce significant mouse body or organ weight loss and did not have any additive toxicity (134). The immunosuppressant FTY720 is a structural analog of sphingosine and is phosphorylated to form FTY720-phosphate by SK2 [reviewed in White et al. (141)]. FTY720 and (S)-FTY720 vinylphosphonate inhibit SK1 catalytic activity (142) and induce its proteasomal degradation (143). In MCF-7 breast cancer cells, FTY720 prevented S1P-stimulated rearrangement of actin (144). Monotherapy with FTY720 demonstrated limited efficacy as a single modality therapy (120), and superior efficacy was seen when FTY720 was combined with doxorubicin (119) and subtherapeutic doses of docetaxel (123). Therefore, reduction of SK1 activity in cancer cells by SK1 inhibitors alone may not be sufficient for cancer treatment. Inhibitors that merely reversibly inhibit enzyme activity can be short acting; the efficacy, duration of action, and induction of apoptosis by these inhibitors may be contributory factors for this insufficiency (144). The SK1 inhibitor/chemotherapy combination has proved highly efficacious for overcoming chemotherapeutic resistance and chemosensitization. When this approach is applied via nanocarriers, a superior targeting approach with minimal toxicity can be achieved (123).

## Conclusions and Future Perspectives

Analysis of the literature illustrates that SK1 has a clear role in breast cancer development, progression, and spread, while SK1 knockdown can reverse these processes. In breast cancer cells, SK1 has been shown to interact with various pathways involved in cell survival and chemoresistance, such as NFκB, Notch, Ras/MAPK, PKC, and PI3K. SK1 is upregulated by estrogen signaling, which, in turn, confers cancer cells with resistance to tamoxifen. S1P produced by SK1 has been shown to upregulate the expression of MMP-9 resulting in an invasive phenotype. Both SK1 and S1P are closely linked to inflammation and adipokine signaling in breast cancer. In human tumors, high SK1 expression has been linked with poorer survival and prognosis. SK1 is upregulated in triple negative tumors and basal-like subtypes. It is often associated with high phosphorylation levels of ERK1/2, SFK, LYN, AKT, and NFκB and high expression of S1PR1, 3, and 4. The relationship between SK1 and HER2 is more complex, and careful patient stratification and/or choice of SK1 assessment (RNA vs. protein) may be critical for meaningful clinical correlation. Higher tumor SK1 mRNA levels were correlated with poor response to chemotherapy.

There is ample evidence that SK1 inhibition has significant therapeutic potential. SK1 inhibitors have been shown to reduce breast cancer cell proliferation, clonogenic survival, migration, and invasion. Importantly, a better outcome has been achieved in combination with other therapies such as gefitinib or docetaxel. Therefore, SK1 may have a potential as a target for precision medicine, its high expression being a negative prognostic marker in ER-negative breast cancer, as well as a target for chemosensitizing therapy.

There are four key areas in the field of SK1/breast cancer biology/therapy that, in our opinion, may have the greatest potential for yielding clinically meaningful data.

1. Further assessment of the role of SK1/S1P in the tumor microenvironment. It is well-known that the immune system plays an enormous role in cancer detection/clearance. A recent publication in Cell Reports (145) has outlined the crucial role of S1P in lymphocyte differentiation from memory toward a regulatory (inhibitory) phenotype, suggesting that local S1P depletion may be instrumental in re-educating the immune system.
  In breast cancer, the tumor microenvironment plays a key role in both tumor initiation and progression. Obesity-related chronic inflammation, secretory adipokines, and fat-derived estrogens are all known to predispose to tumor development and we now have evidence of the role of SK1 in these settings (120, 133). It is possible that by targeting SK1 in these environments, we can move further into cancer prevention rather than treatment of late-stage cancer, when it may be too late. Additionally, a dietary approach and the use of natural substances as mild SK inhibitors might be considered. A number of natural products with SK inhibitory activity have been isolated from different sources [extensively reviewed in (146, 147)].
2. Further identification of the exact role of SK1 expression in disease progression. Studies looking at the expression of RNA, protein, or phosphorylated protein showed conflicting results. Two important issues are often overlooked in such studies: (a) the fact that SK1 is an enzyme and it is the enzymatic activity that in the end determines its pathophysiological role, and (b) the exact location of measured expression—in some studies, tumor tissue was not differentiated from stroma and fat tissue, all of which are present in the breast microenvironment. In addition to SK1 expression, its role in expression/activity of other oncogenic factors may be further explored (148, 149).
3. Clarification of the potential role of SK1 inhibitors in cancer therapy. Initially, pan-SK inhibitors were often used alone, and their efficacy was assessed in comparison to chemotherapy. With the development of more selective SK1 inhibitors, it transpired that some of these compounds did not cause cell death, while achieving good levels of SK1 inhibition (137). With the dominating hypothesis that high SK1 levels are required for tumor development and growth, these data puzzled researchers and prompted some to suggest that SK1 inhibitors may have low therapeutic value. It is possible, however, that in many tumors, high SK1 levels confer additional proliferative/anti-apoptotic benefits, but are not required for cell survival. If this is true, the use of SK1 inhibitors as adjuvants to chemo- or radiotherapy may be more beneficial than their use as monotherapy.
  Additionally, prolonged SK1 inhibition generates a wide genetic response, including upregulation of multiple prosurvival pathways as well as expression of SK2, which provides cells with missing S1P (149). This again warrants a trial evaluating the use of SK1 inhibitors in combination with other molecular therapy or chemotherapy (122, 150), or alternatively one utilizing pan-SK inhibitors, rather than selective SK1 inhibitors.
4. Use of nanocarriers in delivering combination therapies. There is now evidence that nanoparticle-based therapies are advantageous due to characteristics such as targeted drug delivery and precise kinetics of release (123, 151). Additionally, nanocarriers may confer other properties, including imaging capability and minimization of toxicity, when used for delivery of combined therapies.

## Author Contributions

HA, HT, and DP wrote and reviewed the manuscript. DP developed the idea.

### Conflict of Interest

The authors declare that the research was conducted in the absence of any commercial or financial relationships that could be construed as a potential conflict of interest.
